# Can Smoking Cause Differences in Urine Microbiome in Male Patients With Bladder Cancer? A Retrospective Study

**DOI:** 10.3389/fonc.2021.677605

**Published:** 2021-06-08

**Authors:** Wenchao Ma, Wentao Zhang, Liliang Shen, Ji Liu, Fuhang Yang, Niraj Maskey, Hong Wang, Junfeng Zhang, Yang Yan, Xudong Yao

**Affiliations:** ^1^ Department of Urology, Shanghai Tenth People’s Hospital, Tongji University, Shanghai, China; ^2^ Shanghai Clinical College, Anhui Medical University, Hefei, China; ^3^ Department of Urology, The Affiliated People’s Hospital of Ningbo University, Ningbo, China

**Keywords:** smoke, bladder cancer, microbiome, urine, male

## Abstract

**Background:**

Tobacco smoking is a carcinogen for many cancers including bladder cancer. The microbiota is involved in the occurrence, development, and treatment of tumors. We explored the composition of male urinary microbiome and the correlation between tobacco smoking and microbiome in this study.

**Methods:**

Alpha diversity, principal component analysis (PCA) and Adonis analysis, linear discriminant analysis (LDA) coupled with effect size measurement, and PICRUSt function predictive analysis were used to compare different microbiome between smokers and non-smokers in men.

**Results:**

There were 26 qualified samples included in the study. Eleven of them are healthy controls, and the others are from men with bladder cancer. Simpson index and the result of PCA analysis between smokers and non-smokers were not different (P > 0.05) in healthy men. However, the abundance of Bacteroidaceae, Erysipelotrichales, Lachnospiraceae, Bacteroides, and so on in the urinary tract of smokers is much higher than that of non-smokers. Compared to non-smokers, the alpha diversity in smokers was elevated in patients with bladder cancer (P < 0.05). PCA analysis showed a significant difference between smokers and non-smokers (P < 0.001), indicating that tobacco smoking plays a vital role in urinary tract microbial composition.

**Conclusion:**

The composition of microbiome in the urinary tract is closely related to tobacco smoking. This phenomenon is more significant in patients with bladder cancer. This indicates tobacco smoking may promote the occurrence and development of bladder cancer by changing urinary tract microbiome.

## Introduction

Bladder cancer (BCa) is one of the most common carcinoma ranking nine among all cancers (1). Studies have shown that the incidence of bladder cancer has continued to rise since 1990 (2). According to cancer statistics 2020 in America, new cases of bladder cancer are 81,400, and deaths of bladder cancer are 17,980 (3). In general, painless hematuria is the main clinical feature of bladder cancer (4). Pathological examination following transurethral resection of bladder tumor (TURBT) and cystoscopy are the gold standard for diagnosing bladder cancer (5). However, TURBT and cystoscope are both invasive procedures. Urine exfoliative cytology is also used for the diagnosis of bladder cancer while its sensitivity is low (6, 7). Therefore, it still needs further research on the early diagnosis of bladder cancer.

Epidemiological evidence shows that nearly 1/3 of patients with bladder cancer have a history of smoking (8). Cigarette exposure is the most important independent risk factor for bladder cancer and is closely related to the occurrence and progression of bladder cancer (9). The results of a multicenter case–control study showed that the risk of bladder cancer among smokers was higher than that of non-smokers (10). With the increase in the number of cigarettes and the prolongation of smoking age, the risk of bladder cancer has increased significantly (11). The disease-specific mortality rate of smokers who continue to smoke after the diagnosis of bladder cancer is 1.53 times that of those who quit smoking after the diagnosis of bladder cancer (12). These phenomena may be due to aromatic amines and polycyclic aromatic hydrocarbons in cigarette smoke which can cause DNA damage in the urinary tract epithelium and cause changes in the urine environment (13). Previous study also demonstrated that smoking cessation can reduce the risk of occurrence and recurrence of bladder cancer (11).

In recent years, a lot of evidence has shown that microbiome is closely related to human cancers including genitourinary cancers (14, 15). The microbiota of the genitourinary system are the causative factors or cofactors of urinary system tumors such as prostate cancer (16) and bladder cancer (15). The composition and abundance of the patient’s urinary flora vary with urinary system diseases. Studies have found that the microorganisms in the bladder are involved in the development of bladder cancer and play an important role in the treatment (17–19). For example, Bacillus Calmette–Guerin (BCG) directly instilled in the bladder is widely used to prevent recurrence of bladder cancer through inducing and enhancing immune response (20). However, whether smoking will affect the microbiome in the bladder is still unknown. In this study, our aim is to report the composition of the urine microbiome of male patients with bladder cancer and to explore the effect of smoking on urinary tract microbes.

## Materials and Methods

### Materials

All urine samples were collected from Shanghai Tenth People’s Hospital from 2019 to 2020. The inclusion criteria were as follows: 1) all samples were negative standard urine culture before sequencing; 2) patients with bladder cancer were diagnosed by pathology and healthy controls were outpatients whose bladder ultrasound and urine exfoliative cytology were negative; 3) all samples are clean midstream urine. And the exclusion criteria were as follows: 1) individuals with urinary system diseases in the past three months, such as urinary stones, infections, inflammations and cysts; 2) individuals who have used antibiotics in the past three months; 3) individuals with transurethral procedures in the past three months; 4) individuals with other malignant tumors. The samples were collected by specialized biotechnologists and then stored at −80°C until further processing. Finally, 26 urine samples were successfully sequenced by 16S RNA.

### Methods

#### Bioinformatic Analysis

The original image data files obtained by high-throughput sequencing are converted into original sequencing sequences by base calling analysis, which is called raw data. The original paired-end sequencing data undergoes a series of quality control to obtain a relatively high-quality sequence, named valid tags. The valid tags are classified by operational taxonomic unit (OTU), and the most abundant sequence is selected as the representative sequence. Alpha diversity (Simpson index and species richness), beta diversity (principal component analysis), Adonis analysis, and linear discriminant analysis (LDA) coupled with effect size measurements were used to analyze the microbiome difference between groups. Phylogenetic investigation of communities by reconstruction of unobserved states’ (PICRUSt) function predictive analysis was used to compare differential function pathways between smokers and non-smokers in men. Continuous variables and categorical variables were analyzed using student t-test and chi-square test, separately. P value <0.05 is considered a significant statistical difference.

## Results

### Microbial Composition and Diversity of Urine Samples in Healthy Men and Men With Bladder Cancer

**Figure 1:**
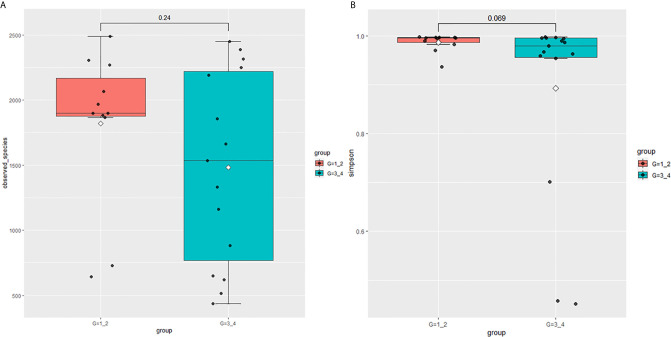
Microbial alpha diversity of urine samples. **(A)** Observed Species, **(B)** Simpson Index.

**Figure 2:**
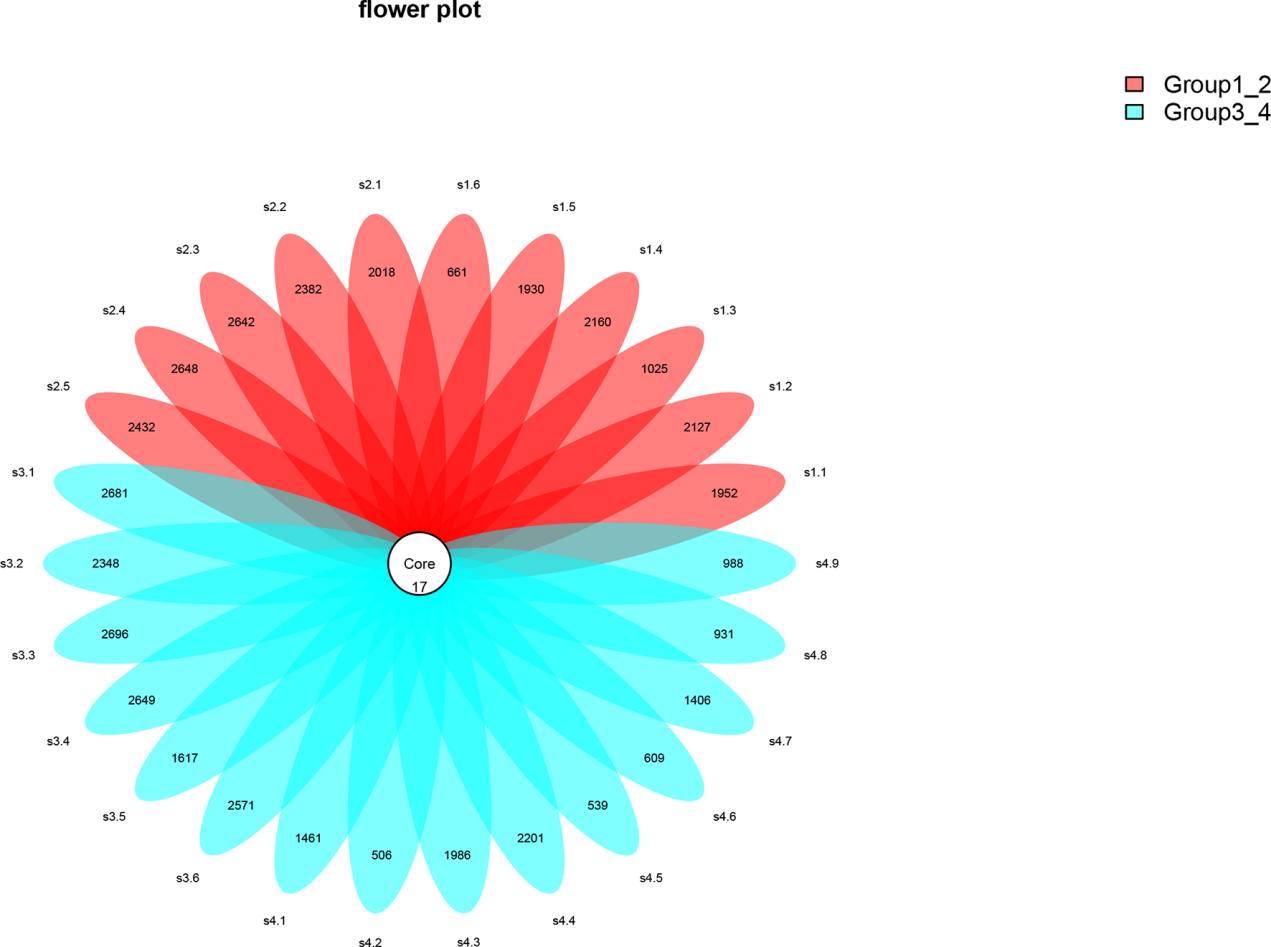
The number of OTUs in 26 samples.

This study performed 16sRNA sequencing on 26 qualified clean midstream urine samples in total, of which 15 urine samples were from men with BCa, and 11 urine samples were from healthy men (**Table 1**). Species richness (P = 0.24) and Simpson index (P = 0.069) reflecting microbial alpha diversity were not different between the two groups (**Figure 1**). However, there are still differences in the number and richness of OTU between samples. The number of OTU is between 506 and 2696 in 26 samples (**Figure 2**). The average OTU of healthy people and those with bladder cancer are 1,998 and 1,679, respectively, and 17 OTUs are present in all urine samples (**Supplementary Table S1**). These microorganisms are likely inherent flora in the urinary tract.

**Table 1:** Clinicopathological characteristics of the samples.

Variables	Healthy men		P value	Men with Bca		P value
	G1	G2		G3	G4	
Age	60.8	61.2	ns	61.8	61.2	ns
BMI	28.7	28.3	ns	27.4	28.1	ns
T stage						ns
NMIBC	\	\		3	4	
MIBC	\	\		3	5	
Grade						ns
Low drade	\	\		2	4	
High grade	\	\		4	5	
Smoke	No	Yes		Yes	No	

NMIBC, Non-muscle-invasive Bladder Cancer; MIBC, muscle invasive Bladder Cancer. ns, no significance; \ , not appplicable.

**Figure 3:**
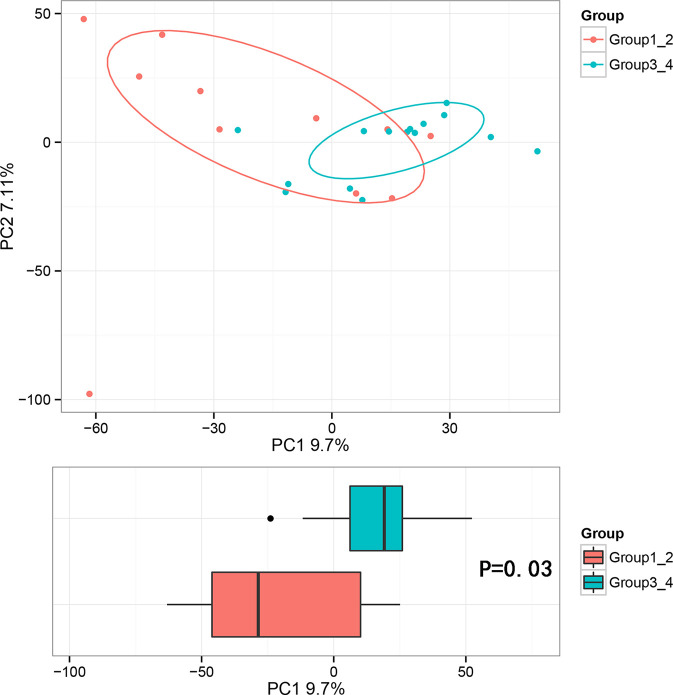
Principal component analysis (PCA) based on OTU abundance between healthy men and BCa patients (Adonis test, Bray–Curtis). x-axis, 1st principal component and Y-axis, 2nd principal component; 9.7% in brackets represents contributions of PC1 components to samples, 7.11% represents contributions of PC2 components to samples. A dot represents each sample, and different colors represent different groups (red: healthy men and blue: BCa patients).

Beta diversity analysis was conducted to explore the relation of urine microbiome between healthy men and men with bladder cancer. The PCA results showed that there were significant differences in the microbial composition between the healthy people and bladder cancer patients (P < 0.03, Adonis test, Bray–Curtis, **Figure 3**). The difference species score chart is displayed in **Supplementary Figure S1**. The red bars indicate the species with relatively high abundance in healthy men, and the green bars indicate the species with relatively high abundance in bladder cancer patients. The top five species in bladder cancer patients were *Stenotrophomonas*, *Enterococcaceae*, *Enterococcus*, *Myroides*, and *Parvimonas*. The top five species in healthy controls were Family_XI, Clostridiaceae_1, Sphingomonas, Deltaproteobacteria, and Gemmatimonadetes.

**Figure 4:**
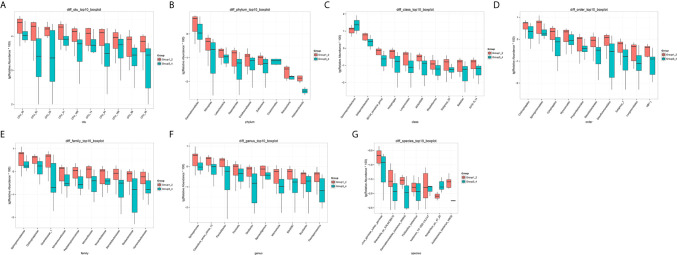
The top 10 different OTU/species at the level of OTU, phylum, class, order, family, genus, and species. **(A)** OTU, **(B)** phylum, **(C)** class, **(D)** order, **(E)** family, **(F)** genus, **(G)** species.

The number of differential OTUs between healthy men and the bladder cancer patients was 498, and the specific distribution in the phylum, class, order, family, genus, and species is shown in **Supplementary Table S2**. The top 10 differential OTU/species at the level of OTU, phylum, class, order, family, genus and species were selected to draw relative abundance boxplot to quickly obtain the abundance of dominant species within groups and difference between groups. There are only nine different phyla between the healthy people and the bladder cancer patients (**Figure 4**). They are Dependentiae, Zixibacteria, Latescibacteria, Halanaerobiaeota, Cloacimonetes, Entotheonellaeota, Rokubacteria, Gemmatimonadetes, and Nitrospirae.

### Effects of Tobacco Smoking to Urinary Tract Microbiome

In order to explore the effects of tobacco smoking on urinary tract microbiome, we performed the subgroup analysis in healthy men and men with BCa, separately.

**Figure 5:**
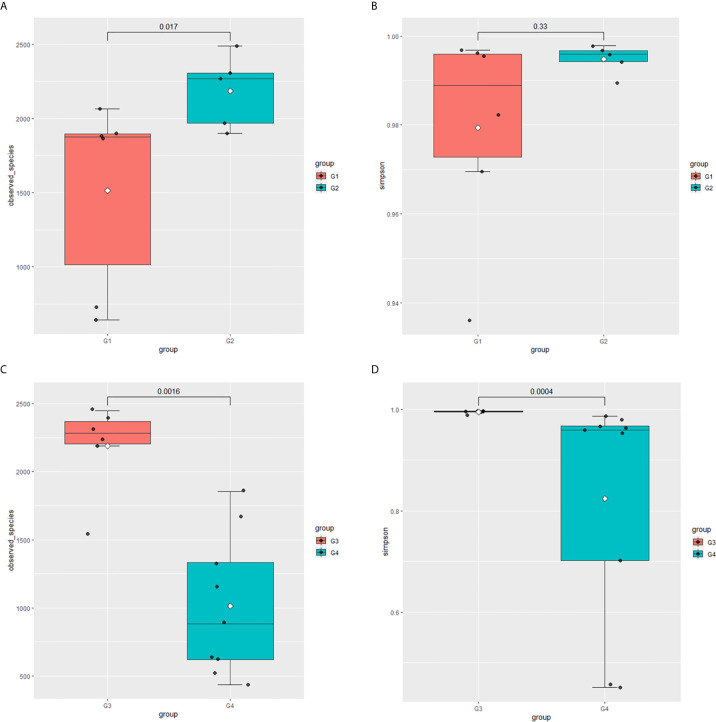
Microbial alpha diversity of urine samples. **(A)** Observed species in healthy men; **(B)** Simpson Index in healthy men; **(C)** Observed species in men with BC; **(D)** Simpson Index in men with BCa.

**Figure 6:**
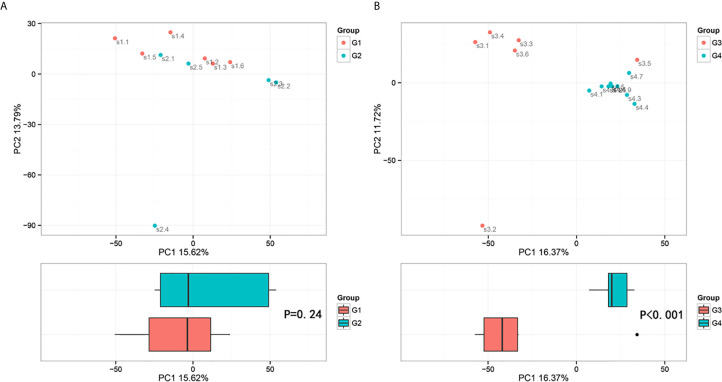
Principal component analysis (PCA) based on OTU abundance (Adonis test, Bray–Curtis). **(A)** Groups 1 and 2, PC1 (15.62%) and PC2 (13.79%). **(B)** Groups 3 and 4, PC1 (16.37%) and PC2 (11.72%).

In 11 healthy men, group 1 (G1) consisted of six non-smokers, while the group 2 (G2) consisted of five smokers. Species richness in G1 was less than that in G2 (P = 0.017, **Figure 5A**). However, Simpson index, which reflects the alpha diversity between two groups, shows no difference (P = 0.33, **Figure 5B**). There was no difference in the results of PCA analysis between G1 and G2 (P = 0.24, **Figure 6**). PICRUSt function prediction analysis was performed based on the 16S RNA sequencing data annotated by the Greengenes database (21, 22). There are 775 differential COGs between smokers and non-smokers (**Supplementary Table S3**), and **Figure 9A** shows the top 30 differential clusters of orthologous groups of proteins (COGs). The results of Kyoto Encyclopedia of Genes and Genomes (KEGG) pathway analysis are displayed in **Figure 10A** at level 3.

**Figure 7:**
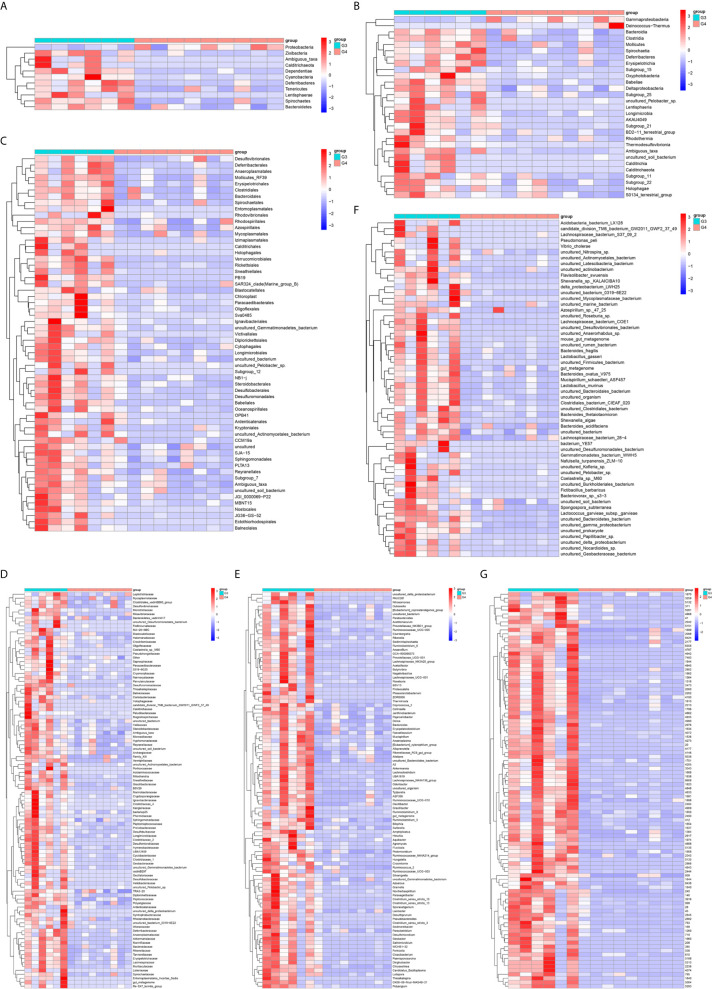
The differential microorganisms at OTU, phylum, class, order, family, genus, and species levels. **(A)** phylum, **(B)** class, **(C)** order, **(D)** family, **(E)** genus, **(F)** species, **(G)** OTU.

**Figure 8:**
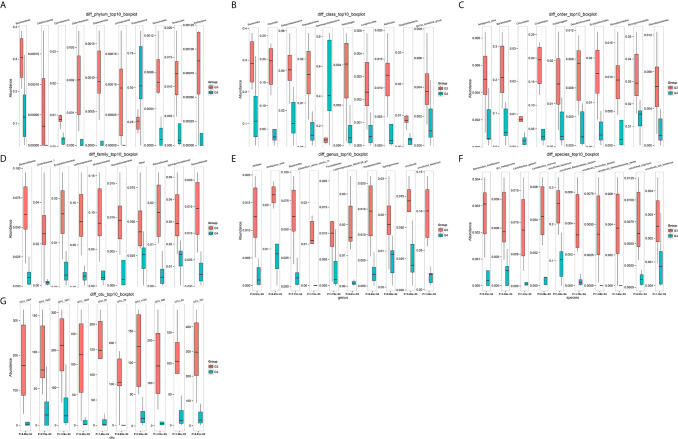
The top 10 differential microorganisms at OTU, phylum, class, order, family, genus, and species levels. **(A)** phylum, **(B)** class, **(C)** order, **(D)** family, **(E)** genus, **(F)** species, **(G)** OTU.

The results in BCa patients were different from those in healthy men. Out of 15 men with BCa, six smokers belong to group 3 (G3), and the rest were non-smoker labeled as group 4 (G4). The species richness and Simpson index in G3 were significantly higher than that in G4 (P < 0.05, **Figures 5C, D**). The PCA analysis which reflects the beta diversity also showed that the microbiome between G3 and G4 has significant differences (P < 0.001, Adonis test, Bray–Curtis, **Figure 6B**). To further explore the characteristics of the urinary tract microbiome in smokers and non-smokers with bladder cancer, we analyzed the differential microorganisms with heatmap at OTU, phylum, class, order, family, genus, and species levels. If the number of differences exceeds 100, only the top 100 will be displayed (**Figure 7**). The top 10 differential microbiota at phylum, class, order, family, genus, species, and OTU levels between G3 and G4 were displayed in **Figure 8**. The top ten differential phylum were Dependentiae, Spirochaetes, Deferribacteres, Zixibacteria, Bacteroidetes, Lentisphaerae, Tenericutes, Cyanobacteria, Proteobacteria, and Calditrichaeota. Furthermore, the difference in species score chart was displayed in **Supplementary Figure S3**. The red bars indicate the species with relatively high abundance in smokers with bladder cancer, and the green bars indicate the species with relatively high abundance in non-smoking bladder cancer patients. The top five species in smokers were Bacteroidetes, Bacteroidia, Bacteroidales, Clostridia, and Clostridiales. The top five species in non-smokers were Proteobacteria, Gammaproteobacteria, Pseudomonadales, Moraxellaceae, and Acinetobacter.

**Figure 9:**
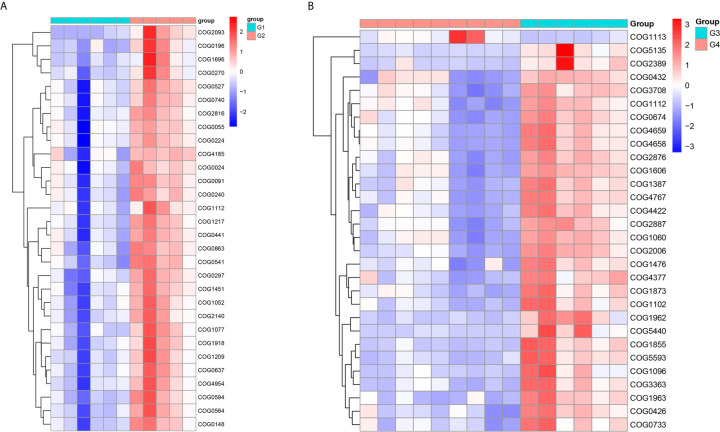
The top 30 differential COG between smokers and non-smokers. **(A)** Differential COG in healthy men; **(B)** differential COG in men with BCa.

**Figure 10:**
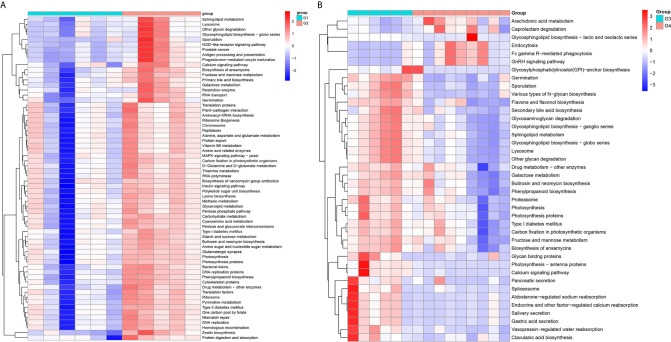
The Kyoto Encyclopedia of Genes and Genomes(KEGG) pathway analysis between smokers and non-smokers. **(A)** Differential pathways in healthy men, **(B)** differential pathways in men with BCa.

We functionally annotate genes by searching against Clusters of Orthologous Groups of protein (COG) database. There are 1,180 differential COGs between smoking and non-smoking bladder cancer patients (**Supplementary Table S3**), and the top 30 differential COGs were displayed in **Figure 9B**. The Kyoto Encyclopedia of Genes and Genomes (KEGG) pathway analysis was also applied to explore function differences between G3 and G4. At level 2, the results showed smokers have significantly higher metabolism than non-smokers in BCa patients. At level 3, there are 40 different pathways between smoking and non-smoking BCa patients, and they are displayed in **Figure 10B** with heatmap.

## Discussion

Currently, tobacco smoking is the most common risk factor for bladder cancer, and it is also associated significantly with the mortality of BCa (23). Smoking increases the risk of bladder cancer by two to four times, and it is correlated positively with the intensity and duration of smoking (24, 25). On one hand, previous study has demonstrated that the expression of DNA methyltransferase 1 (DNMT1) in smokers was significantly higher than that of non-smokers in bladder cancer (26), and the levels of methylated metabolites, polycyclic aromatic hydrocarbons, DNA adducts, and DNA damage were elevated in smokers with bladder cancer. This is because tobacco-specific carcinogens can be transformed into active intermediates interacting with DNA and the intermediates are potentially carcinogenic (27, 28). On the other hand, there are a variety of cigarette smoke metabolites in the smokers’ urine, and the two most abundant metabolites are cotinine and 4-(methylnitrosamino)-1-(3-pyridyl)-1-butanol (NNAL) (29). These metabolites have certain carcinogenic potential. However, the effect of smoking on urinary tract microorganisms has not been studied. Previous studies have shown that urinary tract microbes were closely related to the occurrence and treatment of bladder cancer. For example, BCG vaccine is a live vaccine made from a suspension of attenuated *Mycobacterium bovis* and applied to treat bladder cancer, which can enhance the activity of macrophages and the ability of macrophages to kill tumor cells, activate T lymphocytes, and improve the body’s cellular immunity (30, 31). Alfano M et al. once discussed the important role of extracellular matrix and microflora contributing to tumorigenesis (32).

Previous studies have explored the effects of smoking on microorganisms in some parts of the body. For example, the gut microbes are significantly different between smokers and non-smokers. Compared with non-smokers, smokers have increased abundance of phylum Bacteroidetes and a decrease in Proteobacteria (33). The study by Brotman R. et al. showed that smoking can lead to a decrease in vaginal Lactobacillus in women (34). In genitourinary system, smoking and microbial infections are both high-risk factors for genitourinary tumors. For example, kidney infection and smoking can increase the risk of renal cell carcinoma (35). The study by Shrestha et al. (36) reported that the urinary microbiome in men with prostate cancer may be enriched with proinflammatory bacterial species associated with prostatitis, urinary tract infections, and bacterial vaginosis. Another study also highlighted the role of microorganisms in the progression of prostate cancer (16). A former study by Moynihan et al. (37) found that smoking has no effect on urine microbiota in male with hematuria. There are still some differences in urinary tract microbes between bladder cancer patients and healthy controls (38). However, there are currently no studies focusing on the effect of smoking on urethral microbes in healthy people or bladder cancer patients. The purpose of this study is to explore the effects of smoking on urinary tract microorganisms and find potential therapeutic targets.

Firstly, we explored the effects of smoking on urethral microbes in healthy men. In alpha diversity analysis, the observed species in smokers (G2) were higher than in non-smokers (G1). Simpson index and principal component analysis (PCA) between smokers and non-smokers had no difference. That means the effect of smoking on urinary tract microbes in healthy people is limited. However, there are still some differences between smokers and non-smokers in urinary tract microbes in healthy people. For example, the abundance of microorganisms in the urinary tract of smokers, including Bacteroidaceae, Erysipelotrichales, Lachnospiraceae, Bacteroides, and so on, is much higher than that of non-smokers. Bacteria and Family_XI are more abundant in the urine of non-smokers (**Supplementary Figure S2**). At level 2, the enrichment results of KEGG show that the immune system, translation, nucleotide metabolism, glycan biosynthesis and metabolism, metabolic diseases and nervous system in smokers are more active than in non-smokers in healthy people. Many metabolic pathways and COG in smokers are more active than those in non-smokers.

We also explored the effect of smoking on urinary tract microbes in BCa men. The results show that smoking has an important effect on urinary tract microbiome. The alpha diversity (Observed species and Simpson index) in smokers (G3) with BCa was higher than those in non-smokers (G4). Beta diversity analysis showed that tobacco smoking is the main factor affecting urinary tract microbial composition. The heatmap shows that the abundance of multiple microorganisms in the urine of smokers is higher than that of non-smokers at the phylum, class, order, family, genus, species, and OTU levels, and the details were displayed in **Supplementary Table S2**. At the phylum level, bacteroidetes, zixibacteria, ambiguous_taxa, calditrichaeota, dependentiae, cyanobacteria, deferribacteres, tenericutes, lentisphaerae, and spirochaetes increased and proteobacteria decreased in smokers. This result is similar to the effect of smoking on gut microbiota (33). According to the results of KEGG analysis, the metabolic diseases in smokers are more active than in non-smokers in people with bladder cancer at level 2. These results indicate that the metabolic diseases were higher in smokers both in healthy and BCa patients. In addition, there are 16 pathways that are higher in healthy and BCa smokers, and details were saved in **Supplementary Table S4**.

The microbiota composed of symbiotic bacteria and other microorganisms that inhabited the host epithelial barrier plays a key coordination role in cancer treatment (39). Microbes and enzymes can directly affect chemotherapy drugs by affecting drug absorption and metabolism (40, 41). Moreover, the intestinal microbiota indirectly affects the metabolism of oral and systemic chemotherapy drugs by regulating gene expression and the physiological effects of local mucosal barriers and distant organs (42–46). Immune checkpoint inhibitors, antibodies against cytotoxic T lymphocyte-associated antigen 4 (CTLA4), programmed cell death protein 1 (PD1) or its programmed cell death protein ligand 1 (PDL1), have strong anti-tumor ability in experimental animal models and have shown clinical efficacy in cancers including bladder cancer (47–51). Recent studies have reported that the intestinal flora is also involved in the treatment of cancer with anti-CTLA4 and anti-PDL1 (50, 52). Microbe, BCG, has been used to treat bladder cancer for decades and has good curative effects for immunity enhancement. For example, interleukin 8 (53), pro-inflammatory cytokines, interleukin 6 (54, 55), intracellular adhesion molecule 1, and other chemokines are up-regulated due to the interaction between BCG and urothelial cells, and these immune changes promote the interaction between effector cells and tumor cells (31). Microbial infection and inflammation are risk factors for genitourinary tumors (56). Previous study has suggested that the microbiome in the bladder may promote or inhibit urothelial carcinogenesis by changing the extracellular matrix (32). However, the specific mechanism of microbes involved in the progression of bladder cancer is still unclear. Therefore, it is necessary to study the types of urinary tract microorganisms, the mechanism of action, and the possibility of using the microflora as the target to prevent toxicity and improve the anticancer effect. It is worth mentioning that our research provides new ideas for the study of the mechanism of smoking involved in the progression of bladder cancer.

However, there are some limitations in our study. First, this study was only analyzed in urinary tract microbiome in male patients, and future studies is required in female patients. Second, our study is retrospective. Therefore, it is impossible to observe whether the urethral microorganisms of smokers change after smoking cessation. Third, types of cigarettes, smoking frequency, and eating habits may affect the microbial composition of the urinary tracts. Finally, this study is limited by the sample size, and it is a single center study, so the results may lead to a contingency in some degree. In the future, we hope for a multi-center joint research.

## Conclusion

The microbiome in the urine of healthy men and men with BCa is different. Tobacco smoking may play an important role in the changes of microbiome in the urine. This study fills the gap that smoking may promote the occurrence and development of bladder cancer by changing urinary tract microbes and may provide new ideas for the diagnosis and treatment of bladder cancer.

## Data Availability Statement

The processed data required to reproduce these findings cannot be shared at this time as the data also form part of an ongoing study. Requests to access the datasets should be directed to XY, yaoxudong1967@163.com.

## Ethics Statement

The studies involving human participants were reviewed and approved by Ethics Committee of the Tenth People’s Hospital of Shanghai (SHSY-IEC-4.1/19-120/01). The patients/participants provided their written informed consent to participate in this study.

## Author Contributions

Conceptualization, WM and WZ. Data curation, JZ. Formal analysis, WM. Funding acquisition, XY. Investigation, WM. Methodology, HW. Project administration, XY. Resources, WM and FY. Software, JL. Validation, LS and NM. Visualization, WM. Writing—original draft, WM. Writing—review and editing, WM, WZ, and YY. All authors contributed to the article and approved the submitted version.

## Funding

Clinical Special Project of Shanghai Municipal Health Commission (202040179) Shanghai Science Committee Foundation (19411967700). National Natural Science Foundation of China Youth Project, project approval number: 81802554.

## Conflict of Interest

The authors declare that the research was conducted in the absence of any commercial or financial relationships that could be construed as a potential conflict of interest.

## Abbreviations

BCa, bladder cancer; OUT, operational taxonomic unit; NMIBC, Non-muscle-invasive Bladder Cancer; MIBC, muscle invasive Bladder Cancer; PCA, principal component analysis; LDA linear discriminant analysis; PICRUSt, phylogenetic investigation of communities by reconstruction of unobserved states; COG, clusters of orthologous groups of proteins; KEGG, Kyoto Encyclopedia of Genes and Genomes; DNMT1, DNA methyltransferase 1; CTLA4, cytotoxic T lymphocyte-associated antigen 4; PD1, programmed cell death protein 1; PDL1, programmed cell death protein ligand 1.
