# Supplementary figures and images for: Can Smoking Cause Differences in Urine Microbiome in Male Patients With Bladder Cancer? A Retrospective Study
Source: Front Oncol. 2021 Jun 8;11:677605. doi: 10.3389/fonc.2021.677605 (PMC8217881; doi:10.3389/fonc.2021.677605)

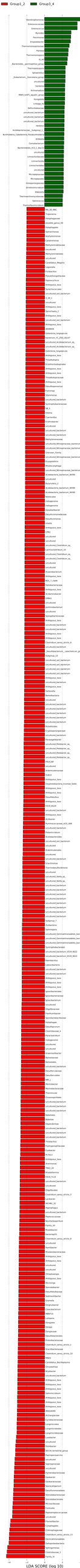

Supplement: Supplementary file 5 [file Image_1.pdf]

G1 G2

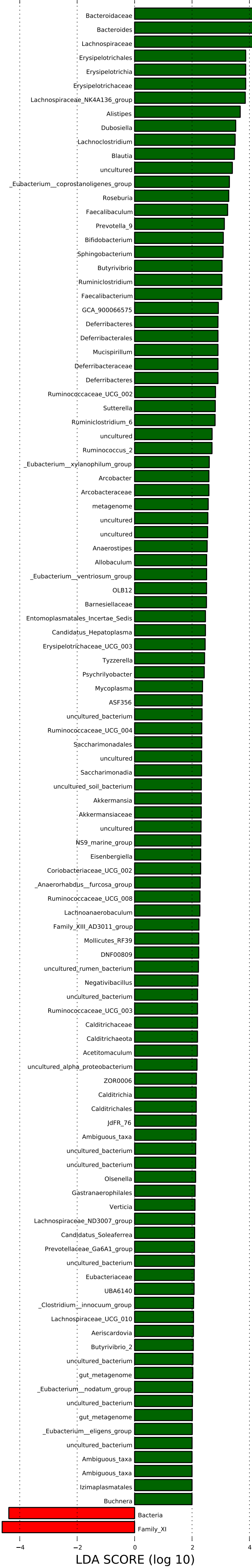

Supplement: Supplementary file 6 [file Image_2.pdf]

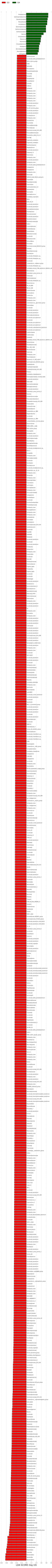

Supplement: Supplementary file 7 [file Image_3.pdf]
